# Supplementary material for: Comparative Analysis of Hepatic Gene Expression Profiles in Murine and Porcine Sepsis Models
Source: Int J Mol Sci. 2024 Oct 15;25(20):11079. doi: 10.3390/ijms252011079 (PMC11507144; doi:10.3390/ijms252011079)
Supplement: Supplementary file 1 [file ijms-25-11079-s001.zip › Supplemental after revision/Supplemental IJMS.pptx]

## Slide 1
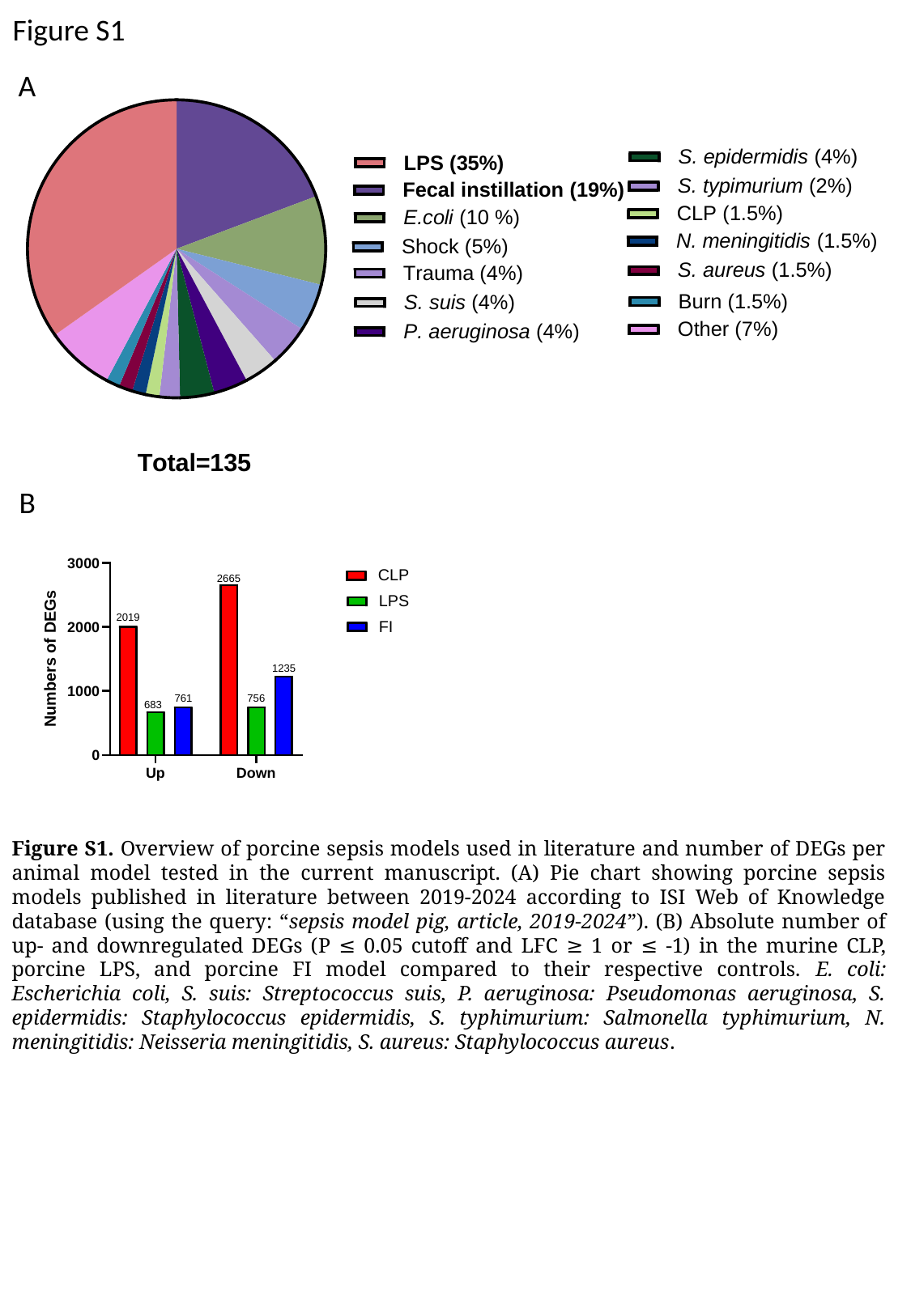

Figure S1
A
B
Figure S1. Overview of porcine sepsis models used in literature and number of DEGs per animal model tested in the current manuscript. (A) Pie chart showing porcine sepsis models published in literature between 2019-2024 according to ISI Web of Knowledge database (using the query: “sepsis model pig, article, 2019-2024”). (B) Absolute number of up- and downregulated DEGs (P ≤ 0.05 cutoff and LFC ≥ 1 or ≤ -1) in the murine CLP, porcine LPS, and porcine FI model compared to their respective controls. E. coli: Escherichia coli, S. suis: Streptococcus suis, P. aeruginosa: Pseudomonas aeruginosa, S. epidermidis: Staphylococcus epidermidis, S. typhimurium: Salmonella typhimurium, N. meningitidis: Neisseria meningitidis, S. aureus: Staphylococcus aureus.

## Slide 2
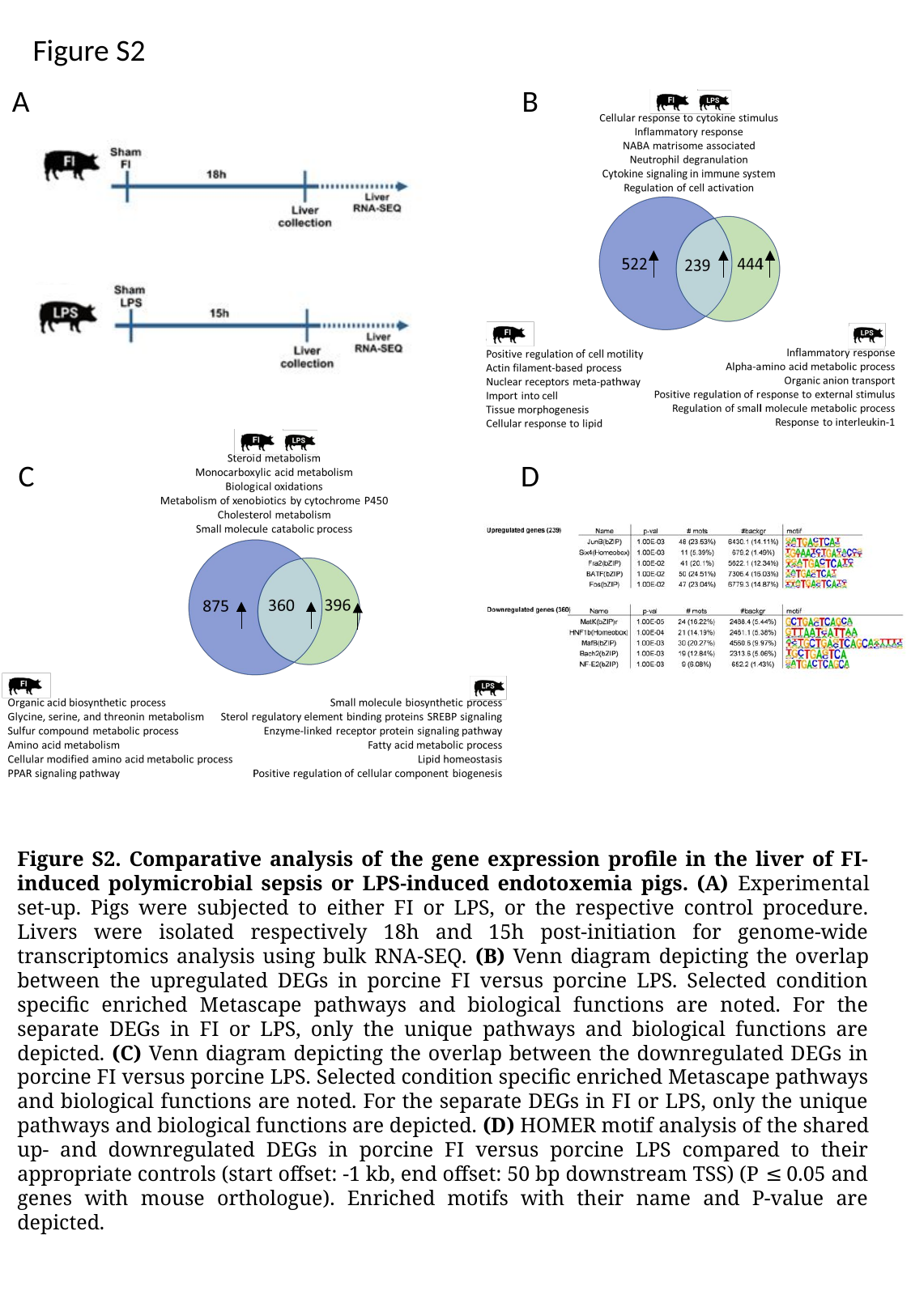

Figure S2
A
B
C
D
Figure S2. Comparative analysis of the gene expression profile in the liver of FI-induced polymicrobial sepsis or LPS-induced endotoxemia pigs. (A) Experimental set-up. Pigs were subjected to either FI or LPS, or the respective control procedure. Livers were isolated respectively 18h and 15h post-initiation for genome-wide transcriptomics analysis using bulk RNA-SEQ. (B) Venn diagram depicting the overlap between the upregulated DEGs in porcine FI versus porcine LPS. Selected condition specific enriched Metascape pathways and biological functions are noted. For the separate DEGs in FI or LPS, only the unique pathways and biological functions are depicted. (C) Venn diagram depicting the overlap between the downregulated DEGs in porcine FI versus porcine LPS. Selected condition specific enriched Metascape pathways and biological functions are noted. For the separate DEGs in FI or LPS, only the unique pathways and biological functions are depicted. (D) HOMER motif analysis of the shared up- and downregulated DEGs in porcine FI versus porcine LPS compared to their appropriate controls (start offset: -1 kb, end offset: 50 bp downstream TSS) (P ≤ 0.05 and genes with mouse orthologue). Enriched motifs with their name and P-value are depicted.

## Slide 3
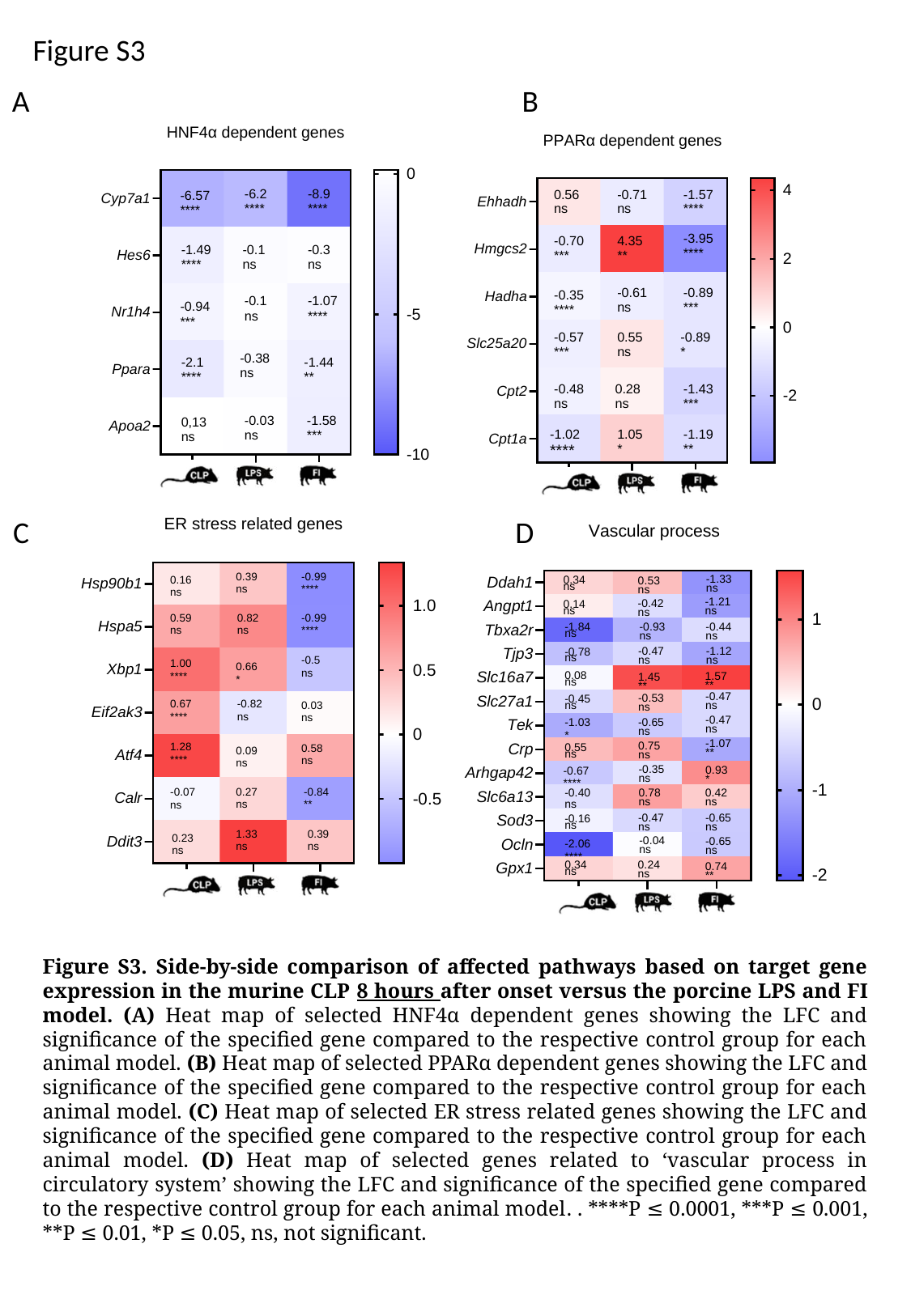

Figure S3
A
B
C
D
Figure S3. Side-by-side comparison of affected pathways based on target gene expression in the murine CLP 8 hours after onset versus the porcine LPS and FI model. (A) Heat map of selected HNF4α dependent genes showing the LFC and significance of the specified gene compared to the respective control group for each animal model. (B) Heat map of selected PPARα dependent genes showing the LFC and significance of the specified gene compared to the respective control group for each animal model. (C) Heat map of selected ER stress related genes showing the LFC and significance of the specified gene compared to the respective control group for each animal model. (D) Heat map of selected genes related to ‘vascular process in circulatory system’ showing the LFC and significance of the specified gene compared to the respective control group for each animal model. . ****P ≤ 0.0001, ***P ≤ 0.001, **P ≤ 0.01, *P ≤ 0.05, ns, not significant.
